# Investigation of the optimum conditions for electricity generation by haloalkaliphilic archaeon *Natrialba* sp. GHMN55 using the Plackett–Burman design: single and stacked MFCs

**DOI:** 10.1186/s12934-022-01810-8

**Published:** 2022-05-13

**Authors:** Ghada E. Hegazy, Tarek H. Taha, Yasser R. Abdel-Fattah

**Affiliations:** 1 National Institute of Oceanography and Fisheries, NIOF-Egypt, El-Anfoushy, Qaitbay Sq, Alexandria, 11865 Egypt; 2 Environmental Biotechnology Department, Genetic Engineering and Biotechnology Research Institute (GEBRI), City of Scientific Research and Technological Applications (SRTA-City), Universities and Research Institutes Zone, New Borg Elarab city, 21934 Alexandria Egypt; 3 Bioprocess Development Department, Genetic Engineering and Biotechnology Research Institute (GEBRI), City of Scientific Research and Technological Applications (SRTA-City), New Borg Elarab city, Alexandria Egypt

**Keywords:** Microbial fuel cell, Archaea, Statistical experimental design, Bioelectricity generation, Stacked MFC

## Abstract

The production of bioelectricity via the anaerobic oxidation of organic matter by microorganisms is recently receiving much interest and is considered one of the future alternative technologies. In this study, we aimed to produce electrical current by using facultative halophilic archaeon *Natrialba* sp. GHMN55 as a biocatalyst at the anode of a microbial fuel cell (MFC) to generate electrons from the anaerobic breakdown of organic matter to produce electrical current. Since the MFC’s performance can be affected by many factors, the Plackett–Burman experimental design was applied to optimize the interaction between these factors when tested together and to identify the most significant factors that influence bioelectricity generation. We found that the factors that significantly affected electrical current generation were casein, inoculum age, magnet-bounded electrodes, NaCl, resistor value, and inoculum size; however, the existence of a mediator and the pH showed negative effects on bioelectricity production, where the maximum value of the 200 mV voltage was achieved after 48 h. The optimum medium formulation obtained using this design led to a decrease in the time required to produce bioelectricity from 20 days (in the basal medium) to 2 days (in the optimized medium). Also, the overall behavior of the cell could be enhanced by using multiple stacked MFCs with different electrical configurations (such as series or parallel chambers) to obtain higher voltages or power densities than the single chambers where the series chambers were recorded at 27.5 mV after 48 h of incubation compared with 12.6 mV and 1.1 mV for parallel and single chambers, respectively. These results indicate that the order of preferred MFC designs regarding total power densities would be series > parallel > single.

## Introduction

MFC is a bio-electrochemical system that drives an electric current using living microorganisms. It is a device that uses microbes to transform the energy stored in the chemical bonds of organic matter into free ions, leading to an electrical current that can be used without the need for combustion [1]. It works by allowing microbes to oxidize organic molecules (anaerobic chamber with anoxic conditions). Microbes at the anode oxidize the organic matter, generating protons that pass through the bridge membrane (which decreases the diffusion of oxygen to the anode) to the cathode, which is immersed in a liquid under aerobic conditions, and the electrons which pass through the anode to an external circuit to generate an electrical current [2]**.** Protons, electrons, and oxygen react to produce water molecules at the cathode [3–5]. Also, the use of MFCs may help in reducing environmental contaminants such as atmospheric carbon dioxide and wastewater, and may also potentially supply a renewable energy source [6].

The understanding of microorganisms' metabolism has increased the reaction’s efficiency [7, 8]. The use of MFCs for electricity production is still not optimized, and the amount of electric current generated by this system is low; however, the potential of such systems is great. Fossil fuel combustion produces a large amount of carbon dioxide, which is a significant greenhouse gas that has shown alarming effects on the climate. In this way, the search for other sources of energy that are eco-friendly and cheap has become an excellent need. MFCs are used as an alternative source that is considered a dependable and clean one without any harmful side-effect [9].

There are several factors affecting the performance of MFCs. These factors include biocatalyst activity (microorganisms), organic matter, electrode reactions, reactor design, and internal resistance [10, 11]. Also, the use of magnets that collect electrons on the anode and the presence of a mediator (phenolphthalein) were used to get better electron movement between oxidative microbial metabolism and the surface of the anode because it works in a pH range of 8.5 to 11, which is similar to that of the alkaline condition of the used haloalkaliphilic archaeon *Natrialba* sp. GHMN55. In addition, the physical and chemical conditions of MFCs are dictated by the nature of the biological part, the essential factor of MFCs. Depending on the growth conditions and the microorganisms, the changes in the external chemical and physical conditions can lead to alterations in many physiological parameters, inhibiting the growth and metabolism of microorganisms used as biocatalysts in the anode, and causing their death [12, 13].

The use of graphite rods from discharged dry cells for the synthesis of electrodes serves two purposes; the first one is waste recycling, which contributes positively to environmental safety, and the second is the low-cost production of MFC electrodes [14].

*Natrialba* sp. GHMN55 is an extremophilic microorganism (halophilic archaea) that belongs to the family Halobacteriaceae. This family contains a group of microorganisms (archaea) that are able to live in extreme habitats such as hypersaline environments with salt concentrations of up to 5 M and high alkalinity (pH up to 12) such as Wadi El-Natrun [15]. The use of an added mediator allowed us to compare both ionic strength conditions; for potential applications, many naturally microbial-synthesized compounds can act as electron carriers.

This study aimed to investigate the performance of halophilic archaeon *Natrialba* sp. GHMN55 used at the MFC anode and the effect of high salt concentrations at the anode compartment, which may increase the media conductivity and improve the MFC’s performance (bioelectricity generation) by decreasing the internal resistance among other studied factors. The Plackett–Burman design was applied to optimize bioelectricity generation from the halophilic archaeon *Natrialba* sp. GHMN55 strain (accession No MW794195) by using 10 factors; casein, pH, NaCl, oxygen, magnet, mediator, resistance, inoculums size, inoculum age, and time.

In this study, we explain that halophilic archaea can be used as biocatalysts in MFCs, and bioelectricity production using these microorganisms is possible and can be enhanced under optimized conditions. The effect of high conductivity on both current production and internal resistance is shown. The new study of the halophilic archaeon *Natrialba* sp. GHMN55 strain and other extreme archaeal physiologies could lead to an increase in the current density and electrical power obtained by MFCs.

## Materials and methods

### Microbial isolation (Source and culture medium)

Sediments and water samples were collected from the Egyptian extreme saltern lake (El- Hamra Lake) Wadi El-Natrun (salinity of up to 4 M NaCl, pH ranging between 9 and 11, and temperatures of up to 40 °C). El-Hamra Lake is located in the Wadi El-Natrun depression in Egypt. Its geographical coordinates are 30º 23ˋ 21̏ North and 30º 20ˋ 45̏ East. It is an alkaline saline lake with an elongated depression of about 90 km northwest of Cairo. Its average length is about 60 km and its average width is about 10 km. The components of the basal medium used in this experimental study were dissolved in distilled water. The pH was adjusted to 11 using NaOH, and the medium was sterilized by autoclaving at 120 °C for 20 min. The composition of the medium was as follows (g/l): Casein, 5; KH_2_PO_4_, 1; MgSO_4_.7H_2_O, 0.2; NaCl, 200; trace metals, 1 ml; and Na_2_CO_3_, 18. The trace metal solution contained (g/l): ZnSO_4_.7H_2_O, 0.1; MnCl_2_.4H_2_O, 0.03; H_3_BO_3_, 0.3; CoCl_2_.6H_2_O, 0.2; CuCl_2_.2H_2_O, 0.01; NiCl_2_.6H_2_O, 0.02; and Na_2_MoO_4_.H_2_O, 0.03. An extremely haloalkaliphilic archaeon named *Natrialba* sp. and coded as GHMN55 was isolated from the El-Hamra Lake, Wadi El-Natrun, Egypt, and was used in this study [15, 16].

### Preparation of inoculums

Pre-cultures of *Natrialba* sp. GHMN55 were prepared by growing them for 3 days (OD_600_ of 0.4 ~ 1 g/L) and 6 days (OD_600_ of 0.95 ~ 3.6 g/L) during the logarithmic phase in the basal medium broth. Both growing periods were used as the inoculum (10%, (v/v)) in all the experiments unless other percentages are stated [17].

### SEM observations

A cultured broth sample of the *Natrialba* sp. was firstly prepared for electron microscopy. It was coated with a thin gold film using a sputtering device 54 (JFC-1100 E, JEOL, USA) for 12 min. SEM was performed with a JSM 5300 scanning electron microscope (JEOL, USA) at 20 kV [15].

### MFC architecture

#### Single-unit MFC

**Fig. 1:**
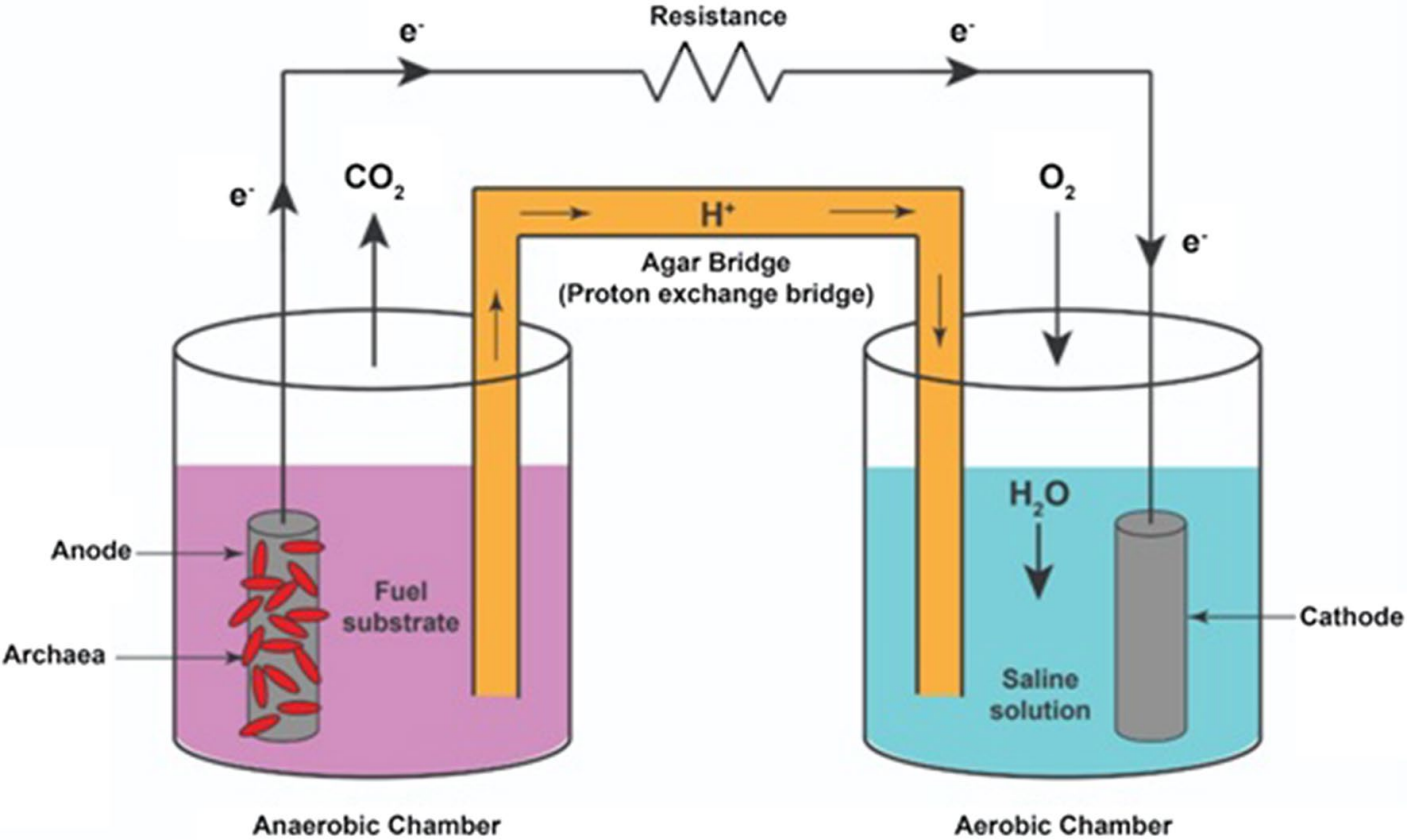
Schematic outline of single-unit MFCs

The applied MFC design was made from cheap and waste materials. Simply, it consists of two cylinder-shaped chambers with a total volume capacity of 300 ml, connected by an agar bridge. Each chamber has a lid with two holes (0.2 and 1.0 cm diameter) that allow the passage of stainless steel connection wires for the electrodes, agar bridge, and the addition of a phenolphthalein mediator. Before inoculation with the microbial biocatalyst, the anolyte solution was covered with mineral oil to inhibit the penetration of oxygen molecules. The catholyte solution was bubbled continuously with air to allow for mixing and increasing the oxygen content of this chamber. Before its use, the cell was sterilized via its overnight immersion into 10% v/v H_2_O_2_, followed by rinsing in sterile distilled water (Fig. 1).

##### Electrodes

Both anodic and cathodic electrodes in the used single-unit MFC were composed of rectangular 3D-graphite bars with dimensions of 6*3*1 cm each. These electrodes with the abovementioned dimensions were used in both anodic and cathodic chambers of both control and experimental MFC units. The graphite bars are used as electrodes in MFC because the graphite̛ structure enables it to be a good electrical conductor. The graphite rods are wrapped with magnetic stir bars measuring 57 mm in length and 27 mm in diameter by wire to condense the electrons. The presence of several electrons enables the rapid generation of electricity through the graphite rods. Before their use, they were cleaned via their immersion for 1 h in 1 mol L^−1^ HCl and NaOH, rinsed, and stored in sterile distilled water [18].

##### Anolyte and catholyte composition

The anolyte was composed of 300 ml of basal broth medium amended with 120 µM of phenolphthalein and inoculated with (10% (v/v)) *Natrialba* sp. while the catholyte was composed of saline solution. The control units were composed of the same experimental unit components but lacked the archaeal inoculation in the anolyte chamber. It is worth mentioning that both anolyte chambers in the experimental and control units were covered by 3 ml of mineral oil to avoid oxygen penetration.

##### External connections and the agar bridge

Both the anode and cathode of the experimental MFC unit were externally connected through 1-mm-diameter stainless steel wires with an intermediate 1000 Ω resistor to close the external circuit. The agar bridge in both experimental and control MFC units was composed of 1.5% agar in U-shaped glass tubes. The agar powder was dissolved in d.H_2_O, poured into the glass tubes, and left to cool down at room temperature for 30 min before use. This bridge was used to connect both the anodic and cathodic chambers of each MFC unit to each other to allow proton transportation between the two chambers.

##### MFC measurement

The MFC analysis and its electrical power measurement were carried out. Current (I) production was calculated using Ohm’s law (I = VR^−1^), where V is the voltage and R is the resistance. Current density, j (mA m^−2^), was calculated as j = IS^−1^, where S is the geometrical (projected) surface area of the anode electrode. Power density, P (mW m^−2^), was calculated as P = IVS^−1^ 19.

##### Optimization of the anolyte composition in MFC using the Plackett–Burman design (PBD)

For the evaluation of the relative significance of 10 variables, the Plackett–Burman experimental design was used according to a previous study (Abdel-Fattah et al., 2007) [20] with some modifications. These factors include growth medium components and other physical parameters. Based on the Plackett–Burman design, each variable was examined at two levels; ʻ − 1ʼ for the low level and ʻ + 1ʼ for the high level. The matrix design of the examined factors was screened in 12 experimental trials. All trials were performed in chambers containing 300 ml of the medium. The output response depended on the measured voltage of each trial using a multimeter instrument.

The Plackett–Burman design is based on a first-order model:

$$ {\text{Y }} = \, \beta 0 \, + \, \sum \, \beta {\text{i xi}}. $$

where Y is the response (the produced voltage), **β**_**0**_ is the model intercept, **β**_**i**_ is the variable calculated, and **x**_**i**_ represents the variable. The Pareto plot demonstrates the results of the Plackett–Burman design since it illustrates the absolute relative significance of variables independent of their nature [21].

### Verification experiment

In this step, the optimization formula was prepared, where the most significant variables were used at their optimum levels obtained from the Plackett–Burman design. Also, the other variables with a negative effect value were fixed at their minimum levels. The purpose of this step was to confirm the results of the Plackett–Burman design and to obtain the basic formula of the optimized medium.

### Stacked MFC

**Fig. 2:**
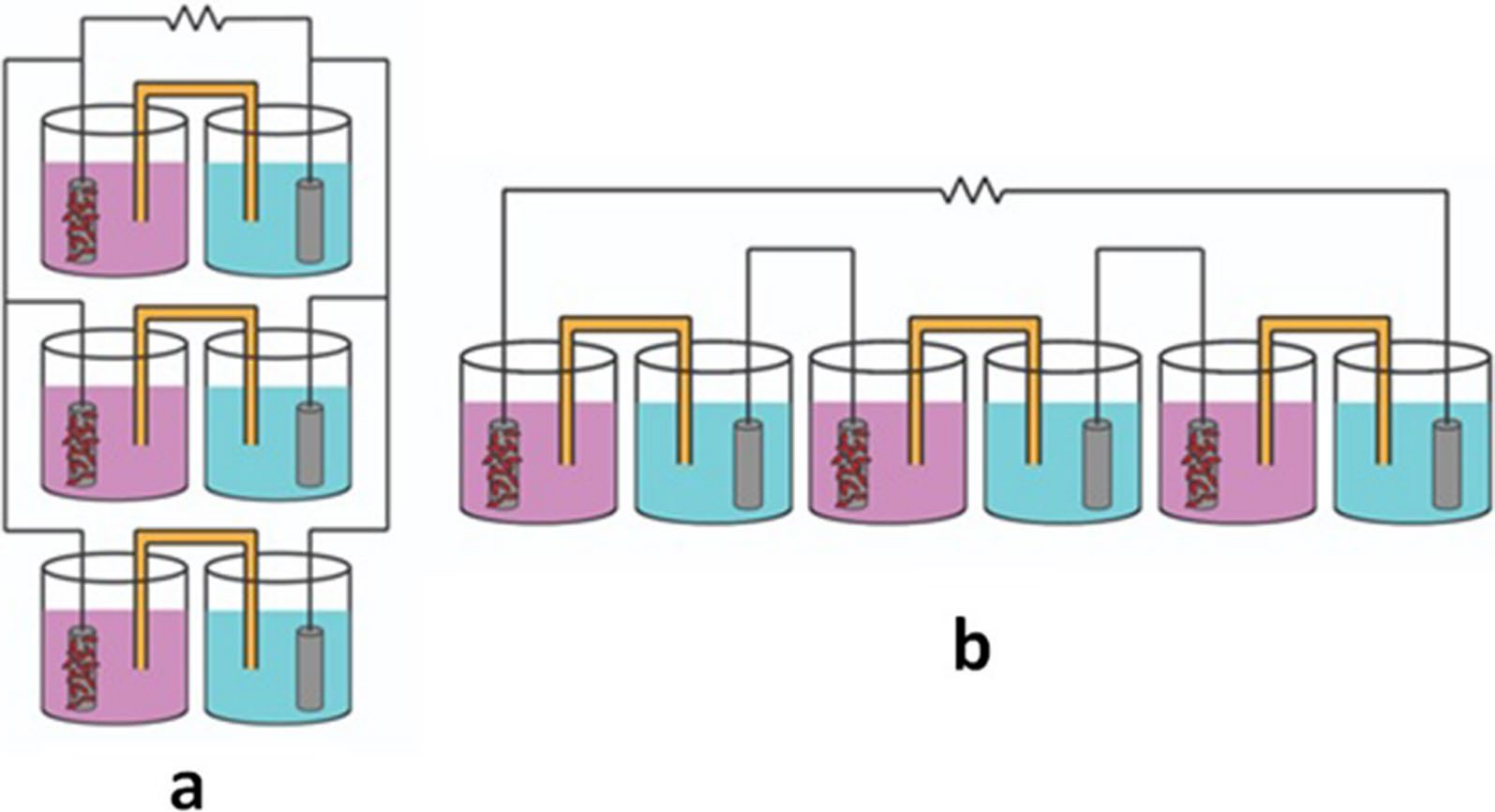
Schematic outline of parallel-stacked MFCs (**a**) and series-stacked MFCs (**b**)

Both parallel and series MFC chambers were separately designed and compared for their overall power behavior with a single MFC. The three units (single, parallel, and series) were the same as all parameters mentioned in the subtitle (a), except that the anodic and cathodic electrodes of all units were different. Each chamber of the three mentioned units included a cylinder graphite electrode that has been previously extracted from waste batteries with dimensions of 6*0.5 cm each and a density of 0.51 g cm^−3^. It is worth mentioning that the optimized components and conditions for this experiment depended on the final recommendations of the statistical design used in the experiment (the Plackett–Burman design). The architecture of the parallel and/or series-stacked MFC chambers is shown in Fig. 2.

### Data from the Plackett–Burman design (PBD) experiments

The BBD data were subjected to multiple linear regressions to estimate the *t*-value, *p*-value, and confidence level. The significance level (*p*-value) was determined using the *t*-test. If this probability was sufficiently small, the idea that an effect was caused by varying the level of the variable under examination was accepted. Optimal values of the dedicated response were estimated using the *solver* function of Microsoft Excel. The simultaneous effects of the three most significant independent factors were generated by *Statistica* 5.0. The optimal conditions obtained were verified experimentally and then compared to the data calculated from the model.

## Results and discussion

### Phenotypic characterization and growth curve of the halophilic archaeon *Natrialba* sp. GHMN55

**Figure3:**
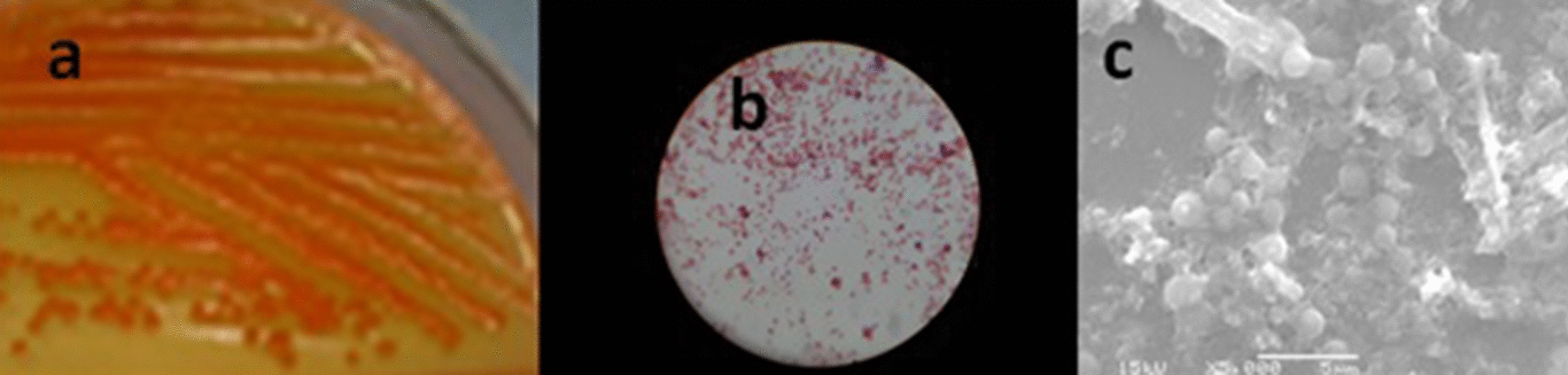
Morphological and microscopic examinations of *Natrialba* sp. GHMN55

**Fig. 4:**
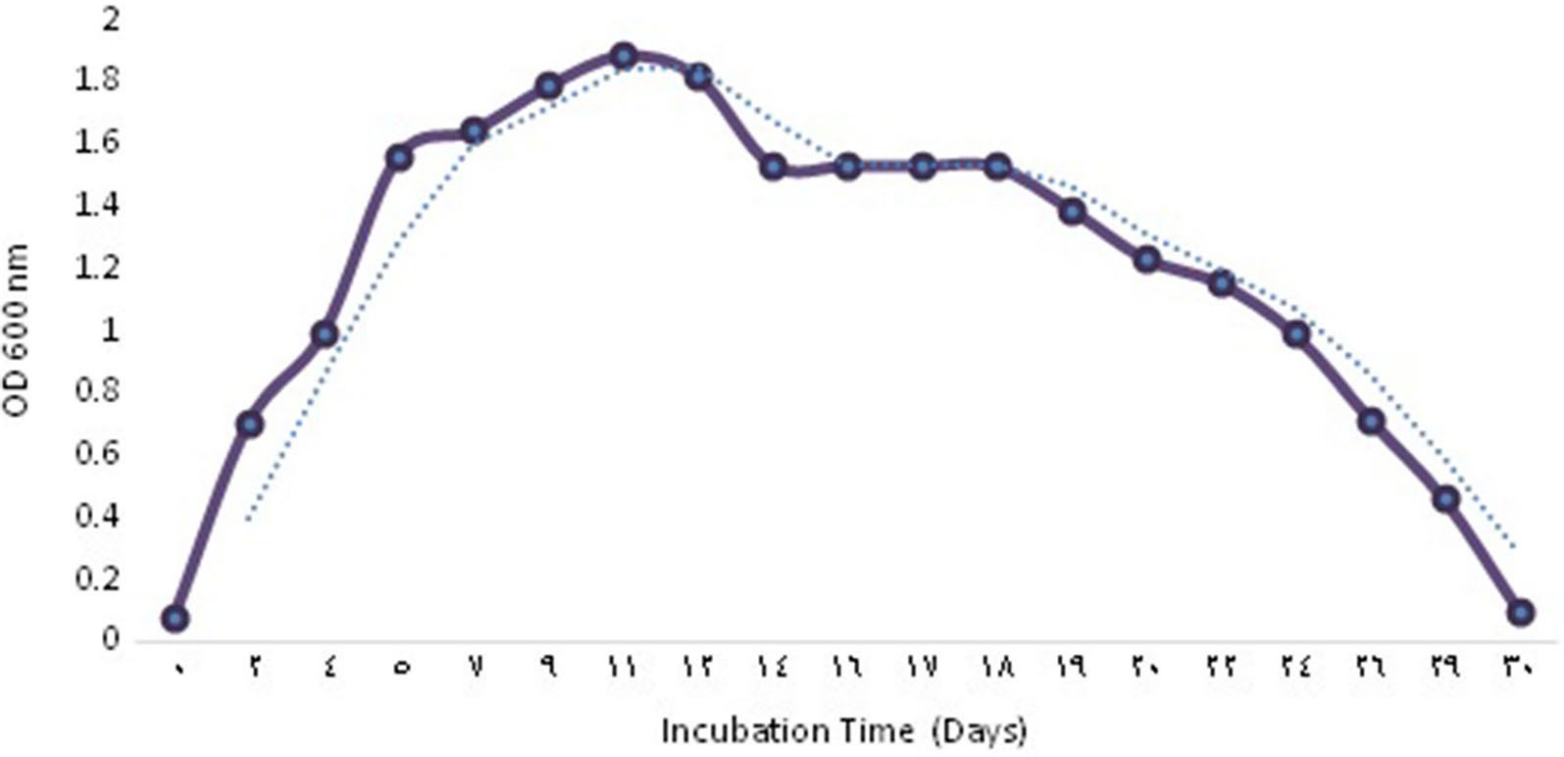
Growth curve of *Natrialba* sp. GHMN55

*Natrialba* sp. GHMN55 (under accession number ac: MW794195) is a Gram-negative bacterium that forms small, round, orange-pigmented colonies when grown on a solid medium after a fifteen-day incubation period at 37˚C (Fig. 3a). Under light and scanning electron microscopes, the cells appeared cocci-shaped (Fig. 3b, c). The growth curve of *Natrialba* sp. GHMN55 is presented in Fig. 4, where a lag phase (adaptation phase where the organism is introduced to new conditions) of four days was observed before the beginning of the log phase. The log phase was continued to the 11^th^ day (the phase in which the utilization of the nutrients and the growth increase rapidly), before the start of the stationary phase (the stage at which the nutrients begin to decrease and the organic matter gets oxidized). After twelve more days, the organism entered the death phase with a gradual decrease in the readings of the optical density [15].

### Power generation performance of MFCs

**Fig. 5:**
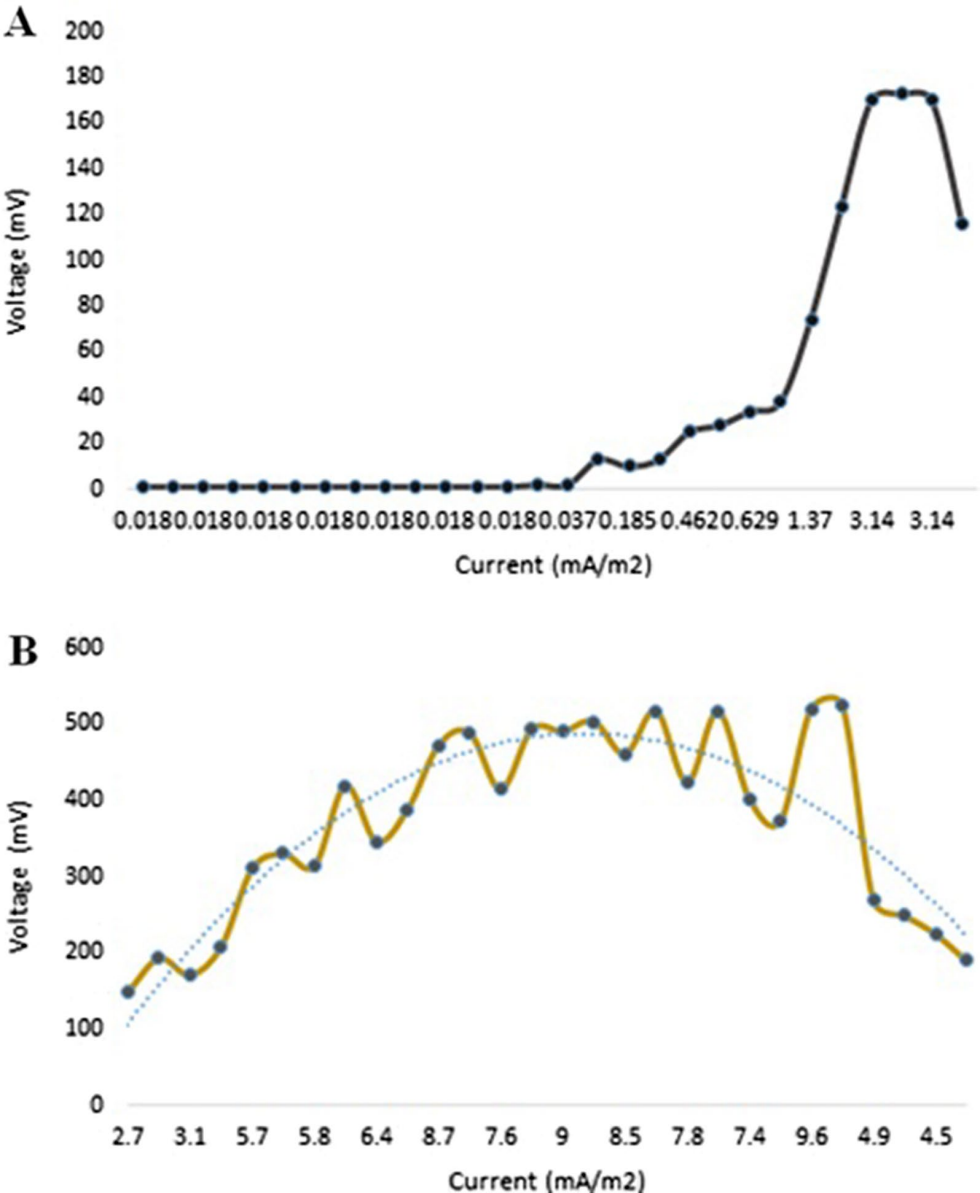
Polarization curves of the designed MFC. **A** the control cell; **B** the treatment cell

**Fig. 6:**
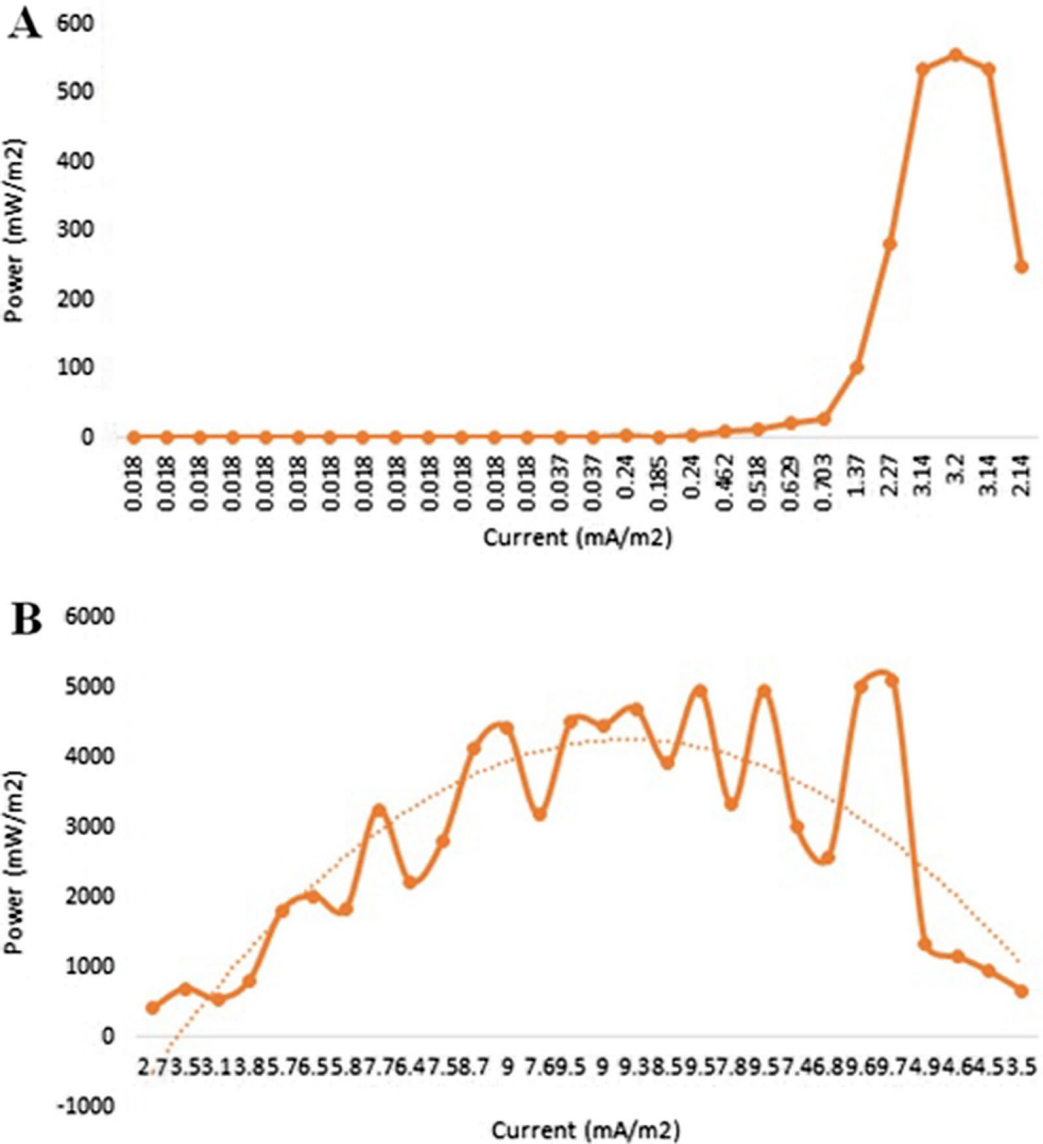
Power density curves of the designed MFC. **A** the control cell; **B** the treatment cell

Figures 5 and 6 show the plots of the polarization and power density curves of both control (without the organism) and treatment MFCs through a prolonged working time (30 days). Upon comparing the control and treatment curves, it can be easily shown that the measured voltage and power are not the same in both cells. The maximum volt reading in the treatment MFC was up to 517 mV after the mid-incubation period (24 days), compared with 13 mV for the control MFC at the same incubation time (Fig. 5). In addition, the power density of the treatment MFC at the mid-incubation time (24 days) was recorded as 4949.7 mW/m^2^, as the maximum reading compared with 3.1 mW/m^2^ for the control MFC at the same time (Fig. 6). We could attribute the production of the maximum voltage or power density after 24 days of incubation to the trend in the typical phases of the tested archaeal growth [22, 23]. These data are strongly matched with the measured growth curve of the tested archaeal strain shown in Fig. 6, where the organism began the death stage and the organic material is consumed and decreased after almost 24 days of incubation. Catal et al. succeeded to produce a maximum power density of 2270 mW/m^2^ when they tested twelve monosaccharides as carbon sources in MFC [24].

On the other hand, after the mentioned mid-incubation time, a gradual voltage drop was observed, reaching 190 mV and 668.5 mW/m^2^ at the end of the experiment for both volt and power density measurements of the treatment MFC. We could attribute this drop to the depletion of the carbon source that would affect microbial behavior. However, it could be suggested that the introduction of an additional carbon source through the experiment would enhance the prolonged stability of the produced power of the cell. This suggestion is strongly matched with the results of Simeon et al., who reported that the continuous production of the electricity from MFC was obtained when the cell was continuously supplemented with the urine as the tested substrate [24].

**Fig. 7:**
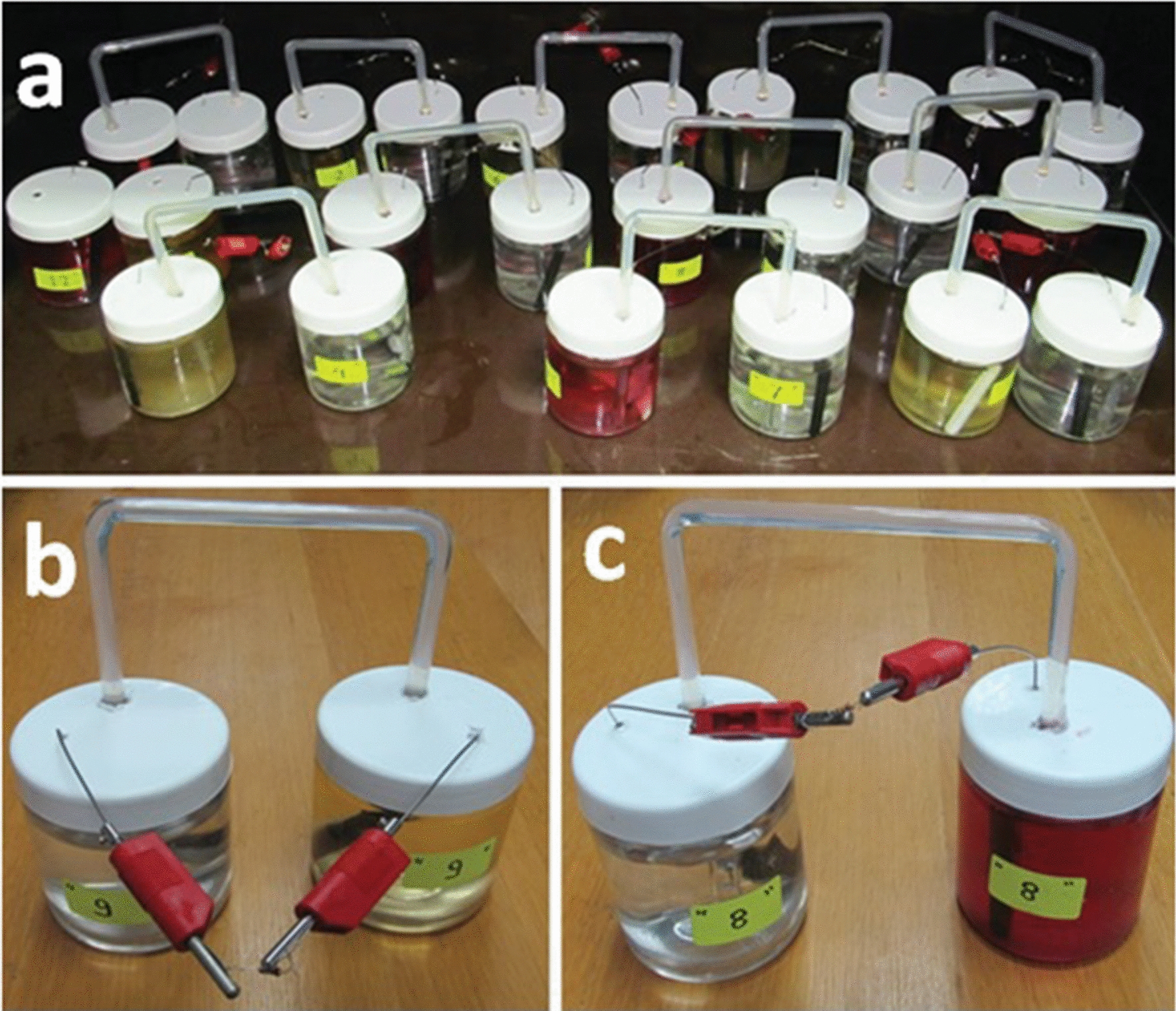
The microbial fuel cell units of the Plackett–Burman design (**a**); single microbial fuel cell without a mediator (**b**); and single microbial fuel cell with a mediator (**c**)

The anolyte medium composition has a very important effect on MFC performance. It is the place where the archaea act as catalysts to anaerobically oxidize the organic substrates present in the anodic chamber and can subsequently produce the electrons and protons that are transferred to the cathode through the wire and the bridge, respectively. The composition of the anolyte depends on the type of the microorganism used in bioelectricity generation. Also, the MFC performance is affected by the ionic strength of the anolyte solution. PBD was used to evaluate the significance of 10 culture factors, including anolyte medium components and other MFC parameters for optimizing bioelectricity production by using haloalkaliphilic archaeon *Natrialba* sp. GHMN55 such as casein, NaCl, pH, inoculum size, culture age, oxygen, magnet, mediator, resistor value, and time. Based on the PBD, each factor was examined at two levels; ʻ − 1ʼ for the low level and ʻ + 1ʼ for the high level. The matrix design of the tested factors was shown in Table 1 and (Fig. 7) in addition to the corresponding results for the tested variables.

### Statistical analysis of the PBD

The PBD involves a linear polynomial correlation model that indicates the correlation between the 10 factors and the response as follows:

Y = 59 + 36 X_1_ + 10X_2_–40.33X_3_ + 2.33 X_4_ + 20.66 X_5_–3.33 X_6_ + 10.5X_7_–22.5 X_8_ + 6.83 X_9_–4.16 X_10_.

**Fig. 8:**
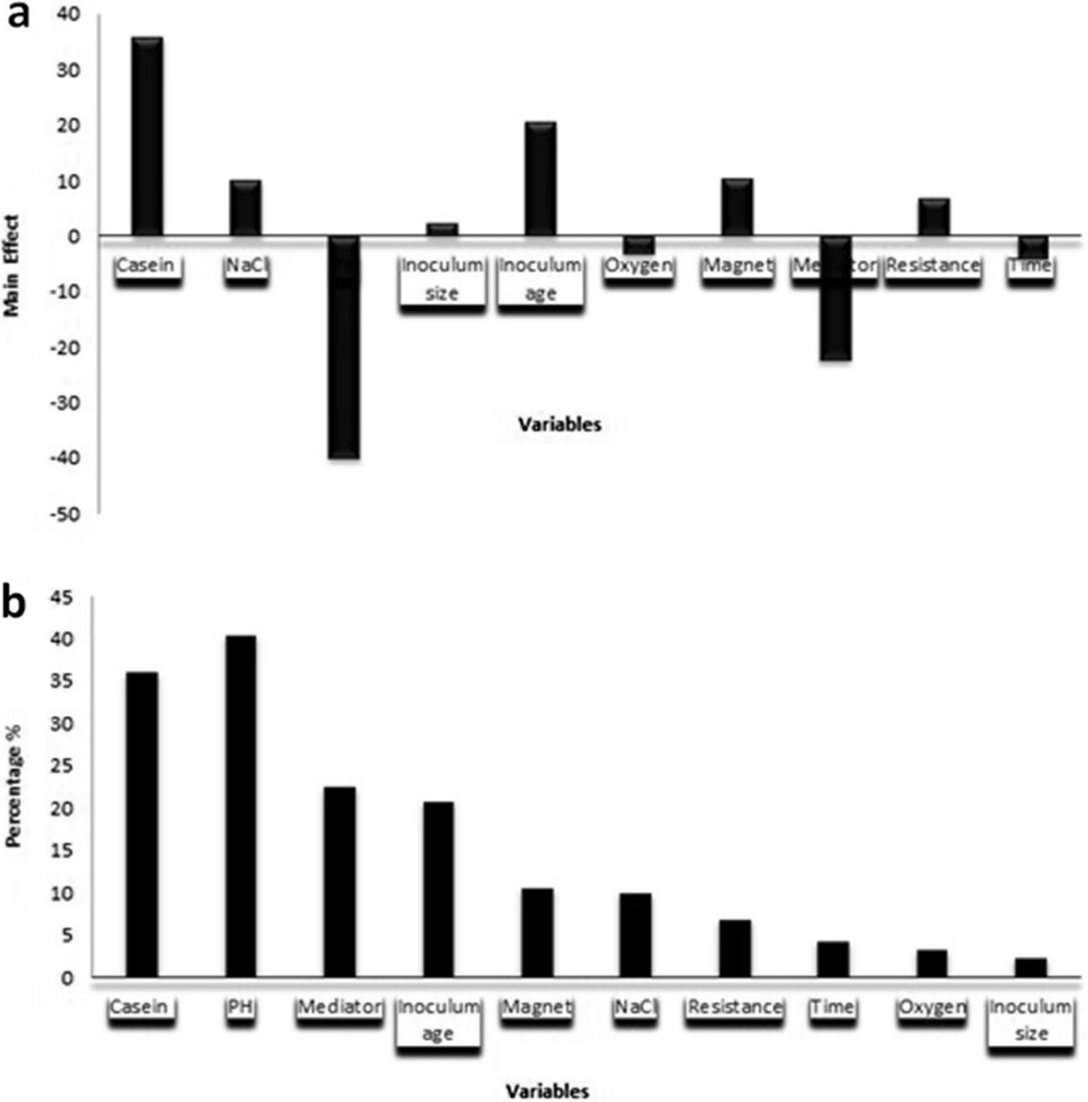
**a** Main effect chart, **b** Pareto chart, rationalizing the effect of each factor on the voltage production by *Natrialba* sp. GHMN55

A large variation in the results of the Plackett–Burman design experiments was recorded, the maximum voltage, 200 mV, was achieved in trial number 10 after 48 h, while the minimum voltage, 0 mV, was achieved in trials number 1, 4, 8, and 11. Based on the main effect in Fig. 8a, the most significant factors affecting bioelectricity production were casein, inoculum age, magnet, NaCl, resistor value, inoculum size, oxygen, time, mediator, and pH, in descending order. By analyzing the regression coefficient R for the ten variables, we found that casein, inoculum age, magnet, NaCl, resistor value, and the inoculum size showed positive effects on bioelectricity production, which means that high concentrations of these variables are near-optimum. On the other hand, time, mediator, and pH demonstrated negative effects on bioelectricity production, which means that low concentrations of these variables are near to optimum, while a main effect near to zero means that this factor has no or little effect on bioelectricity production. The carbon source concentration influences the growth rate of microorganisms and is directly proportional to the MFC performance and is also based on the inoculum size [25]. Previous studies [26, 27] also reported that MFC performance was increased by increasing the glucose concentration used by *Saccharomyces cerevisiae*, indicating that many factors, such as physical (electrode material and resistance), chemical (oxidizing organic material), and biological (the type and age of the used microbe) influence the MFC performance. Another previous study [28] reported that the working of MFCs at a low anodic pH increases the rate of proton transfer and allows for high availability of protons at the cathode surface, which increases electrical current generation.

The Pareto chart has been described as a vital tool for introducing the most important effects (Fig. 8b). It displays the importance of each variable and is a suitable way of examining the results of a Plackett–Burman design. In this graph, the length of each variable bar on a standardized Pareto chart is directly proportional to the value of each calculated variable’s main effect or the regression coefficient.

The t-test for any variable effect tells us the probability that the findings of the calculated effect could have happened by chance or not.

Statistical confidence = (1-*p*)*100.

Where *P* = 0.05 corresponded to a statistical confidence of 95%. So, any variable that showed a statistical confidence interval near or higher than 95% was considered statistically significant.

The accuracy of the model was assessed by the determination coefficient (R2). If the R2 value was calculated to be close to 1.0, the correlation between the calculated and the observed data was considered a very high one; therefore, the present R2 value (0.87) reflected a very good fit between the calculated and observed responses, and it also confirmed that this statistical model was reliable for predicting power generation [29].

### Validation of the model

Based on the obtained analyzed data from Plackett–Burman experimental results, the following composition is predicted to form the optimum medium: Casein, 10 g/l; NaCl, 200 g/l; pH, 10; inoculums size, (10% (v/v)); inoculums age, six days; and resistance, 1000 Ω; with the presence of a magnet and the absence of both mediator and oxygen with incubation time 24 h. To determine the accuracy of the Plackett–Burman design, a verification experiment was performed. The optimum conditions were applied, which gave a power generation with an output voltage of 231 mV.

These results indicated significantly less time required for power generation when compared with those obtained with basal conditions [29].

### Scanning electron microscope

**Fig. 9:**
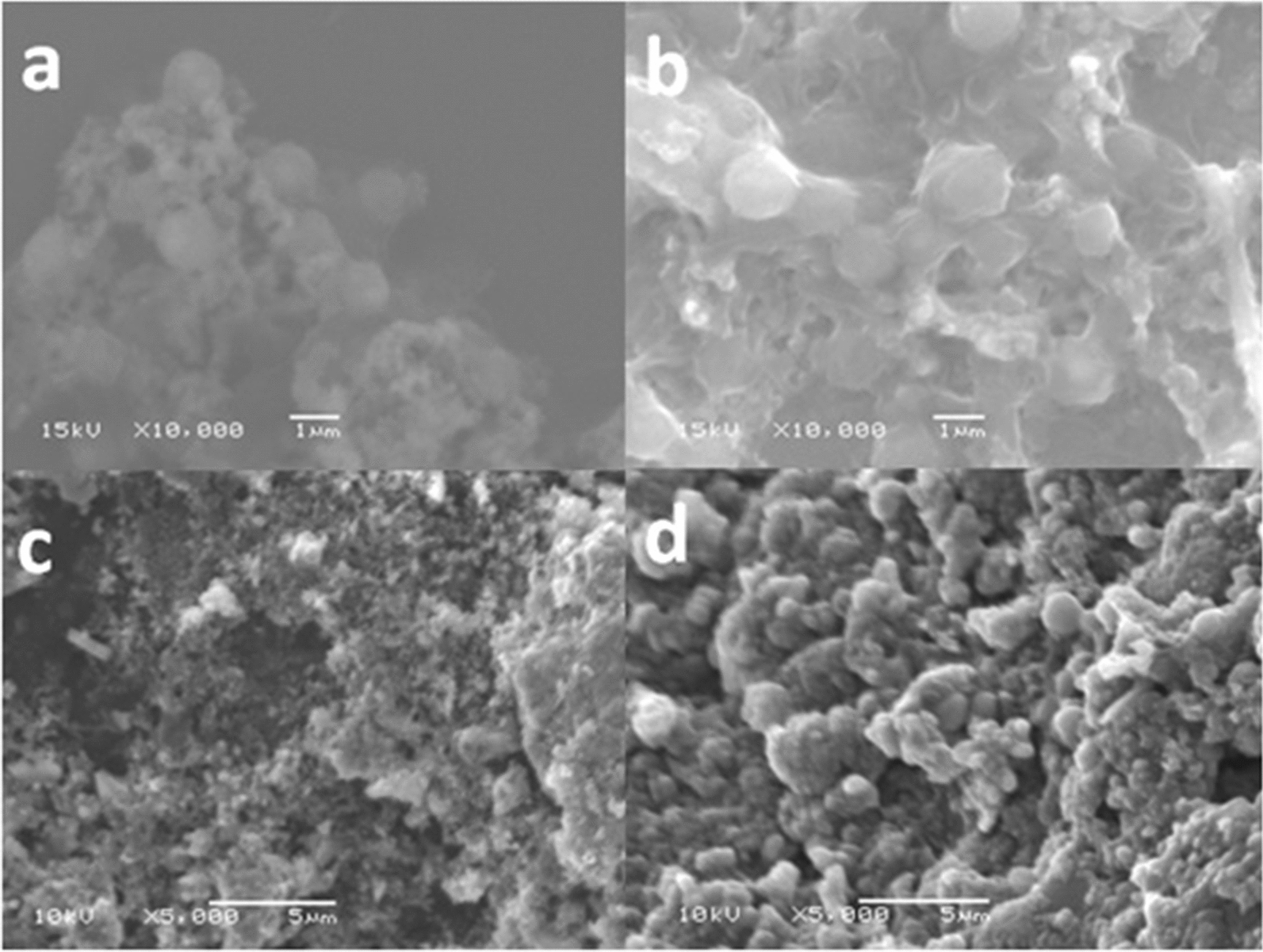
SEM of free and electrode-coating *Natrialba* sp. GHMN55 after three and six days of incubation. **a** Free *Natrialba* sp. GHMN55 cells after three days of incubation, **b** Free *Natrialba* sp. GHMN55 cells after six days of incubation, **c** Archaeal cells–free graphite anodic electrode, and **d** Archaeal cells–bounded graphite anodic electrode

The SEM micrographs of the tested archaeal strain *Natrialba* sp. GHMN55 after three and six days of incubation time, in addition to the anodic electrode in the presence and absence of the archaeal cells, are shown in Fig. 9. The archaeal cells after three days of incubation appeared as smaller cocci cells with a lower cell density (Fig. 9a) compared with the cells after six days of incubation that are shown as larger in size with a higher cell density (Fig. 9b). On the other hand, the micrographs of the cell-free anodic graphite electrode showed that the coarse surface resulted from the attached graphite particles. These un-smooth surfaces may allow the microbial cells to get easily attached to t0he electrode surface and fill in the existent cavities through their biofilm-forming capability (Fig. 9c). This can be easily detected after the co-incubation of the electrode with the microbial cells that showed high cell density incorporation of the cells into the surface cavities of the graphite electrode as shown in Fig. 9d.

### Stacked MFCs

**Fig. 10:**
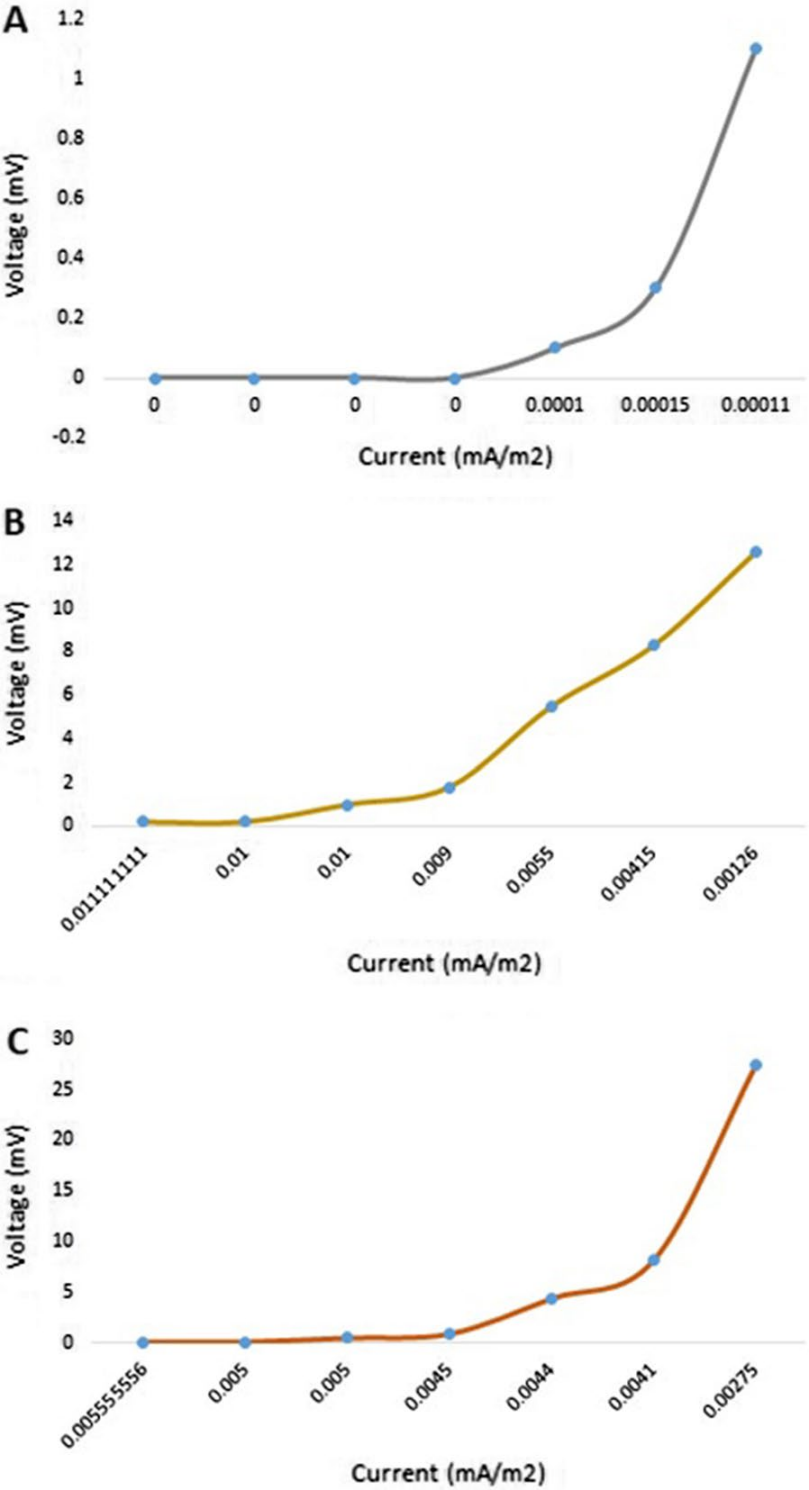
Polarization curves of the stacked MFC. **A** Single-unit, **B** Parallel units, and **C** Series units

**Fig. 11:**
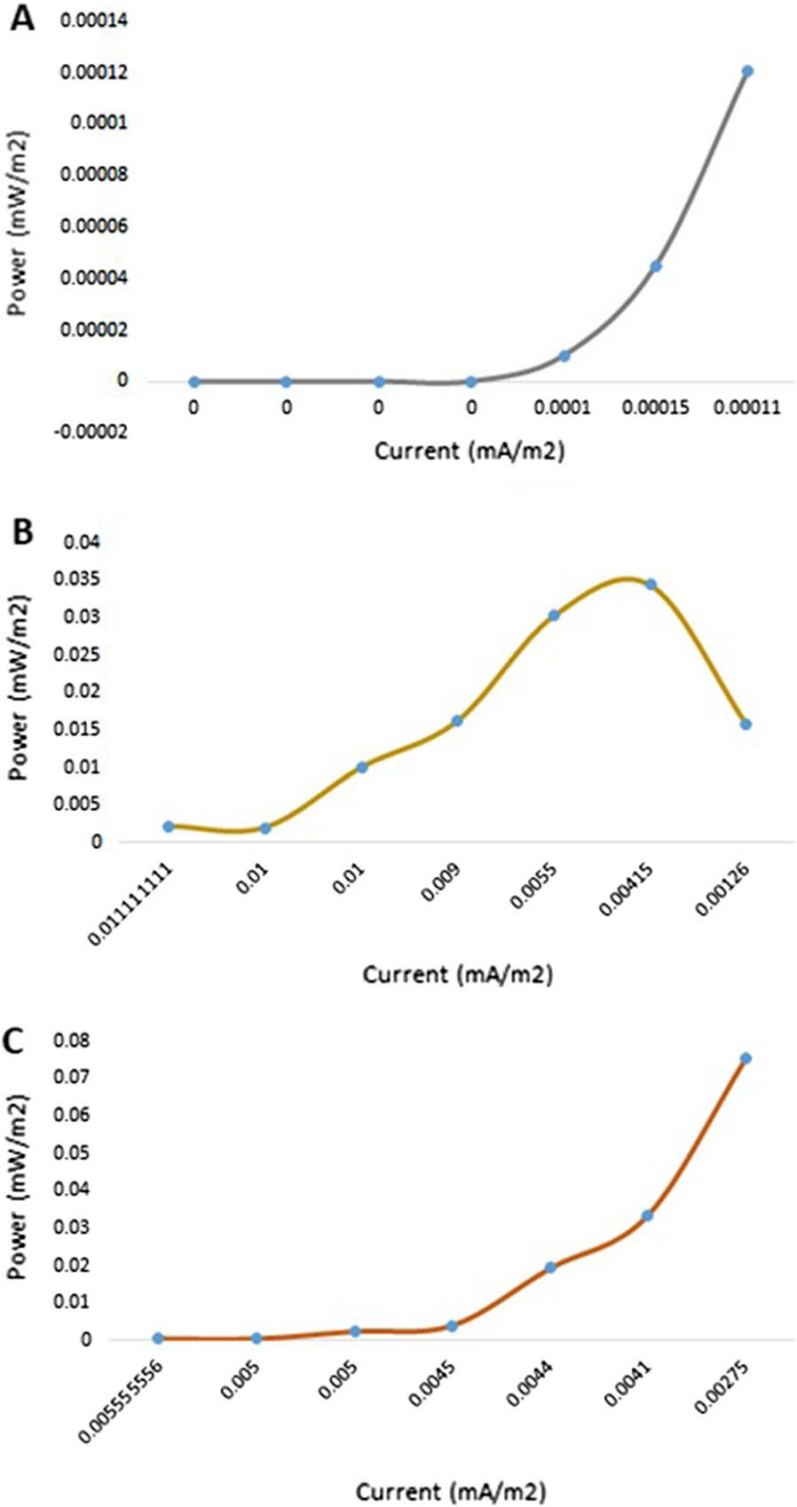
Power density curves of the stacked MFC. **A** Single-unit, **B** Parallel units, and **C** Series units

Increasing the overall produced power by MFCs has been followed by multiple scientists, especially when energy recovery in scaled-up MFC reactors with single chambers was insufficient. To overcome this, multiple stacked MFCs using different electrical configurations (such as series or parallel chambers) were employed to obtain higher voltages or currents [30, 31]. In the current experiment; single, series-stacked, and parallel-stacked MFCs were tested for their voltages and power densities. As shown in Fig. 10, after 48 h of incubation, the measured voltages of all tested chambers (single, parallel, and series) were gradually increased with an increase in the applied resistor (in the range from 90 to 50,000 Ω). It has been observed that the series chambers were able to record 27.5 mV after 48 h of incubation compared with 12.6 and 1.1 mV for parallel and single chambers, respectively (Fig. 10). However, the measured and recorded power densities showed a range of behaviors toward the tested MFC chambers. As shown in Fig. 11, after the same incubation period of 48 h, the parallel chambers showed a maximum power density of 0.034 mW/m^2^ with a resistance of 10,000 Ω, which was reduced to 0.015 mW/m^2^ when a resistance of 50,000 Ω was employed. This behavior significantly differed in the case of single and series chambers. Both of them showed ascending behavior even when a 50,000 Ω resistance was applied, where they recorded 0.0001 and 0.075 mW/m^2^ as the maximum power densities. This indicates that the use of higher resistances would result in higher measured power densities. These results indicate that the order of preferred MFC designs regarding total power densities would be series > parallel > single. Regarding the current density, it followed the same behavior as the power density of the three tested MFC designs. The maximum current densities were recorded as 0.0001, 0.001, and 0.002 mA/m^2^ for single, parallel, and series MFC chambers, respectively, indicating that the parallel design is not the ideal one for the current study. Our results are not in agreement with those of previous investigators [31] who reported that the parallel electrical configuration was preferred over the series configuration when it comes to the produced power. It has been reported that series connections may suffer from contact voltage losses or voltage reversal while parallel ones suffer from increases in the internal losses, which reduces the total power production [32]. We could attribute the lower power densities of the current experiment compared with the single-cell MFC performed at the begging of the current work to the size area of the electrode, which plays an important role in the overall power production [33]. However, Greenman et al. reported in their study that a Square SCMFC utilized leachate from a landfill and showed approximately 75% lower power densities, which could be due to the different inoculants and substrates used when applying a large-scale MFC [34].

## Conclusion

The technology of bioelectricity production can be applied in different modes, including biofuel production, energy production, and bioremediation. Bioelectricity production by microorganisms acts as a sustainable energy source that reduces fossil fuel utilization and leads to the green energy era. Also, the use of wasted batteries as electrode materials that are used here as MFC electrodes can help in recycling waste batteries and, thus, decreasing environmental pollution. The alkaliphilic halophilic archaeon *Natrialba* sp. GHMN55 has been tested for its ability to produce electrical power in laboratory-designed MFCs. The cells have been successfully used to continuously produce current for more than a month, with higher activity at the mid-tested time. The Plackett–Burman experimental design was used to optimize the time and culture conditions required for maximum power production in a minimum time period. The design showed that casein, inoculum age, magnet, NaCl, resistance, and inoculum size had a positive effect on bioelectricity production when used in high concentrations/values while time, mediator, and pH are preferably used in lower quantities. The SEM micrographs showed proper incorporation of the archaea cells into the surface cavities of the graphite electrode. The results of the stacked parallel and series MFCs compared with those of single chambers indicated that both parallel and series MFC chambers are preferred for maximum power production over single cells, while the series design is the ideal one for the current study. We recommend the continuous supplementation of the carbon source in addition to the use of large-area electrodes to gain high power densities and the continuous production of electricity in future designed MFCs.

**Table 1:** Optimization of anolyte solution in Microbial Fuel Cell using statistical experimental design (PBD)

Trials	X1 Casein (g/L)	X2 NaCl (g%)	X3 pH	X4 Inoculum Size (%)	X5 Culture Age	X6 Oxygen	X7 Magnet	X8 Mediator (PP µM)	X9 Resistance (Ω)	X10 Time	Response
										(hrs)	(mV)
**1**	10( +)	15(−)	12( +)	5(−)	3(−)	Nil(−)	P( +)	120( +)	1000( +)	24(−)	0
**2**	10( +)	20( +)	10(−)	10( +)	3(−)	Nil(−)	Nil(−)	120( +)	1000( +)	48( +)	124
**3**	5(−)	20( +)	12( +)	5(−)	6( +)	Nil(−)	Nil(−)	Nil(−)	1000( +)	48( +)	5
**4**	10( +)	15(−)	12( +)	10( +)	3(−)	P( +)	Nil(−)	Nil(−)	100(−)	48( +)	0
**5**	10( +)	20( +)	10(−)	10( +)	6( +)	Nil(−)	P( +)	Nil(−)	100(−)	24(−)	178
**6**	10( +)	20( +)	12( +)	5(−)	6( +)	P( +)	Nil(−)	120( +)	100(−)	24(−)	68
**7**	5(−)	20( +)	12( +)	10( +)	3(−)	P( +)	P( +)	Nil (−)	1000( +)	24(−)	39
**8**	5(−)	15(−)	12( +)	10( +)	6( +)	Nil(−)	P( +)	120( +)	100(−)	48( +)	0
**9**	5(−)	15(−)	10(−)	10( +)	6( +)	P( +)	Nil(−)	120( +)	1000( +)	24(−)	27
**10**	10( +)	15(−)	10(−)	5(−)	6( +)	P( +)	P( +)	Nil(−)	1000( +)	48( +)	200
**11**	5(−)	20( +)	10(−)	5(−)	3(−)	P( +)	P( +)	120( +)	100(−)	48( +)	0
**12**	5(−)	15(−)	10(−)	5(−)	3(−)	Nil(−)	Nil(−)	Nil(−)	100(−)	24(−)	67
Coefficients Main effect *p*-value *t* stat	36 72 0.374 1.5	10 20 0.748 0.416	− 40.3 − 80.6 0.341 − 1.68	2.3 4.6 0.938 0.097	20.6 41.2 0.547 0.861	− 3.3 − 6.6 0.912 − 0.138	10.5 21 0.737 0.437	− 22.5 − 45 0.520 − 0.937	6.8 13.6 0.823 0.284	− 4.1 − 8.2 0.890 − 0.173	– – – –

Note: low level coded (−); high level coded ( +) for the independent variables (X1-X10) presented between brackets are expressed in terms of g (w-/v%) or value. Phenolphthalene (PP), present (P), Not present (Nil)

## Data Availability

All the data generated during this study are included in this published article.
